# Solitary pancreatic metastasis from breast cancer: case report and review of literature

**DOI:** 10.1590/1516-3180.2017.0144260617

**Published:** 2017-11-06

**Authors:** Márcio Apodaca-Rueda, Fábio Henrique Mendonça Chaim, Milena da Silva Garcia, Helena Paes de Almeida de Saito, Martinho Antonio Gestic, Murillo Pimentel Utrini, Francisco Callejas-Neto, Elinton Adami Chaim, Everton Cazzo

**Affiliations:** I Medical Student, Faculdade de Medicina da Pontificia Universidade Católica de Campinas (PUC-Campinas), Campinas (SP), Brazil; II MD. Resident Physician, Department of Surgery, Faculdade de Ciências Médicas da Universidade Estadual de Campinas (FCM-UNICAMP), Campinas (SP), Brazil; III MD. Assistant Lecturer, Oncology Unit - Department of Internal Medicine, Faculdade de Ciências Médicas da Universidade Estadual de Campinas (FCM-UNICAMP), Campinas (SP), Brazil; IV MD, MSc. Assistant Lecturer, Department of Surgery, Faculdade de Ciências Médicas da Universidade Estadual de Campinas (FCM-UNICAMP), Campinas (SP), Brazil; V MD, MSc. Assistant Professor, Department of Surgery, Faculdade de Ciências Médicas da Universidade Estadual de Campinas (FCM-UNICAMP), Campinas (SP), Brazil; VI MD, MSc, PhD. Full Professor, Department of Surgery, Faculdade de Ciências Médicas da Universidade Estadual de Campinas (FCM-UNICAMP), Campinas (SP), Brazil; VII MD, PhD. Adjunct Professor, Department of Surgery, Faculdade de Ciências Médicas da Universidade Estadual de Campinas (FCM-UNICAMP), Campinas (SP), Brazil

**Keywords:** Breast neoplasms, Pancreas, Neoplasm metastasis, Pancreatic neoplasms, Carcinoma, ductal, breast

## Abstract

**ABSTRACT:**

**CONTEXT:**

Pancreatic metastases from primary malignant tumors at other sites are rare, constituting about 2% of the neoplasms that affect the pancreas. Pancreatic metastasis from breast cancer is extremely rare and difficult to diagnose, because its clinical and radiological presentation is similar to that of a primary pancreatic tumor.

**CASE REPORT:**

A 64-year-old female developed a lesion in the pancreatic tail 24 months after neoadjuvant therapy, surgery and adjuvant radiation therapy for right-side breast cancer (ductal carcinoma). She underwent distal pancreatectomy with splenectomy and left adrenalectomy, and presented an uneventful outcome. The immunohistochemical analysis on the surgical specimen suggested that the lesion originated from the breast.

**CONCLUSION:**

In cases of pancreatic lesions detected in patients with a previous history of breast neoplasm, the possibility of pancreatic metastasis should be carefully considered.

## INTRODUCTION

Pancreatic metastases from primary malignant tumors at other sites are rare, constituting about *2%* of the neoplasms that affect the pancreas.^1^ In most cases, the involvement occurs through hematological and lymphatic dissemination, as in cases of kidney and lung carcinomas. It can also occur through contiguous invasion of neighboring organs such as the liver, stomach and spleen. Pancreatic metastasis from breast cancer is extremely rare and difficult to diagnose, because its clinical and radiological presentation is similar to that of a primary pancreatic tumor.^2^^,^^3^^,^^4^^,^^5^ The objective of the present study was to report on a case of pancreatic metastasis of breast cancer, along with the treatment that was proposed.

## CASE REPORT

A 64-year-old female underwent neoadjuvant chemotherapy consisting of doxorubicin, cyclophosphamide and paclitaxel, with subsequent quadrantectomy and axillary lymph node dissection due to a right-side breast neoplasm. Histopathological examination revealed a ductal carcinoma classified as T2N2M0, consisting of a 4-cm tumor with spreading to six axillary lymph nodes but without distant spreading to bones, liver, brain or lungs). It was triple-negative, for estrogen, progesterone and human epidermal growth factor receptor 2 (HER2) receptors. Radiation therapy was subsequently implemented. The patient was then followed up with serial investigations (mammogram, bone scintigraphy scan and computed tomography scans of the cranium, thorax and abdomen) for locoregional and distant relapses every six months.

Twenty-four months after receiving the diagnosis, she evolved with a complaint of left-flank pain, inappetence and loss of seven kilograms in four months. She presented dyspeptic symptoms characterized by early satiety and pain in the upper abdomen after feeding. On physical examination, the abdomen was painful to deep palpation. There was no evidence of relevant laboratory abnormalities.

**Figure 1.**
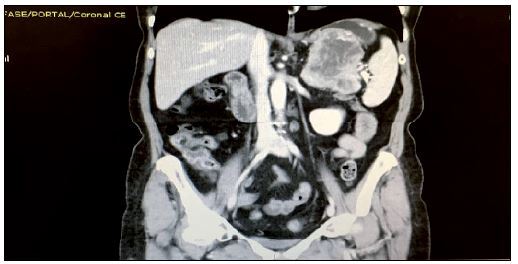
Computed tomography showing a lesion in the tail of the pancreas.

Abdominal computed tomography demonstrated a hypervascularized solid lesion of 6.6 cm x 6.0 cm x 7.0 cm in the tail of the pancreas. It had an irregular outline and partially defined borders, presented a central area of necrosis and was in contact with the anterior margin of the spleen and greater gastric curvature. It was not possible to determine any cleavage plane. A small amount of free liquid was present (**Figure 1**). Cancer antigen (CA)-19.9, carcinoembryonic antigen (CEA) and CA-125 levels were within the normal ranges. No other sites with suspected lesions were detected through positron-emission computed tomography.

**Figure 2.**
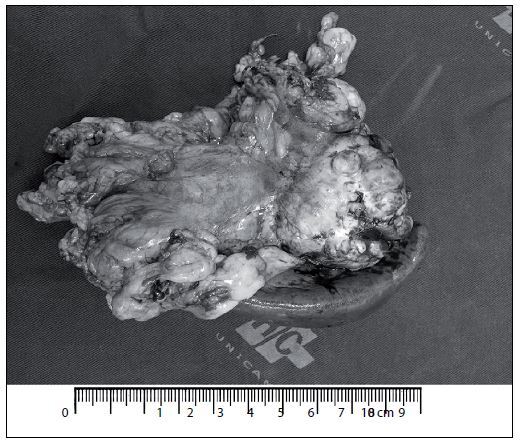
Surgical specimen (distal pancreatectomy with splenectomy and left adrenalectomy).

Because the hypothesis of pancreatic neoplasia needed to be clarified and no endoscopic ultrasound-guided biopsy was available, prompt surgery was warranted given that there was no evidence of other sites of active disease. The patient underwent distal pancreatectomy with splenectomy and left adrenalectomy (**Figure 2**), with uneventful postoperative outcomes. She had good evolution in the postoperative period, with complete remission of symptoms.

The histopathological diagnosis consisted of metastasis from breast carcinoma. The results from the immunohistochemical analysis were positive for the cytokeratin-7 (CK7) marker and negative for the mucin 5AC (MUC-5AC), CEA, CA-19.9, estrogen receptor (ER), progesterone receptor (PR) and Breast-2 (BRST-2) markers. Although negativity for ER, PR and BRST-2 does not favor a breast origin, these markers do not preclude this origin. On the other hand, negativity for the MUC-5AC, CEA and CA-19.9 markers does not favor a pancreatobiliary origin and favors the breast as the primary site. A chemotherapy regimen consisting of paclitaxel was administered for 12 weeks following the patients recovery from the operation, and currently she is being followed up with serial screenings for locoregional and distant spreading of disease every six months. As of 18 months after the diagnosis was made, there is no evidence of active disease.

## DISCUSSION

Breast cancer causes metastases especially to bones, liver and lungs. Pancreatic involvement in solitary metastases from a primary breast neoplasm is rare, occurring in less than 3% of the cases. A review of the literature was conducted through an online search for the Medical Subject Headings (MeSH) terms “breast neoplasms”, “pancreas” and “neoplasm metastasis” in MEDLINE (via PubMed) and LILACS (via BVS) (**Table 1**). We included original studies that reported single cases or case series of this disease or correlated conditions. All the papers were checked according to their titles and abstracts (screening). Full papers were obtained from journals available on the website of the Commission for Improvement of Higher Education Personnel (Comissão de Aperfeiçoamento de Pessoal de Nível Superior, CAPES) (Ministry of Education, Brazil). Unavailable articles were requested from their authors. Articles presenting potentially relevant studies were read and analyzed to assess the inclusion criteria. We excluded articles that consisted of *in vitro* or animal studies, articles in which the participants’ characteristics did not match those mentioned above, poster session abstracts, review articles and other types of publications. Other papers were used for contextualization and discussion.

**Table 1.** Database search results for pancreatic metastasis arising from primary breast cancer

Electronic databases	Search strategies	Results
MEDLINE (PubMed)	(Breast neoplasms) AND (Pancreas) AND (Neoplasm Metastasis)	17 case reports 6 case series
LILACS (BVS)	(((Breast neoplasms) OR (Neoplasias da mama) OR (Neoplasias de la mama)) AND ((Pancreas) OR (Pâncreas) OR (Páncreas)) AND ((Neoplasm Metastasis) OR (Metástase Neoplásica) OR (Metástasis de la Neoplasia)))	1 case report

**Figure 3.**
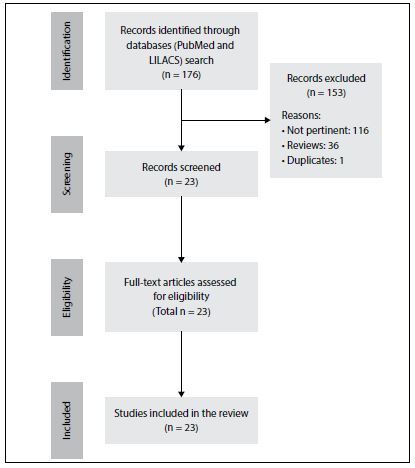
Flow diagram of the review of the literature.

After extensive online research, we identified 23 studies, 17 case reports and 6 case series, totaling 28 reported cases of pancreatic metastases from breast cancer. **Table 2**^2^^,^^3^^,^^6^^,^^7^^,^^8^^,^^10^^,^^11^^,^^12^^,^^13^^,^^14^^,^^15^^,^^16^^,^^17^^,^^18^^,^^19^^,^^20^^,^^21^^,^^22^^,^^23^^,^^24^^,^^25^^,^^26^^,^^27^ summarizes the main articles found and their reported outcomes. **Figure 3** presents a flow diagram of the articles selected. In the majority of the cases described, spreading to the head of the pancreas was more common than to the tail, and the most common histological type was lobular carcinoma; the predominant metastatic pattern was solitary. The average interval between the diagnoses of primary breast neoplasm and pancreatic metastasis was 43.3 months.^2^ In our case, the patient presented metastasis to the region of the tail of the pancreas, with a histopathological diagnosis of ductal carcinoma, and the asymptomatic interval was 24 months.

**Table 2.** Reported cases of pancreatic metastases arising from primary breast cancer

Authors	Breast cancer subtype	Age (years)	Disease-free interval (months)	Presenting symptoms	Location of metastases at diagnosis	Profile of the metastatic disease at diagnosis	Clinical management	Overall survival (months)
Akashi et al.^6^	Lobular	47	41	NR	Head of pancreas	Solitary	Pancreaticoduodenectomy	28
Azzarelli et al.^7^	Lobular	49	43	Jaundice	Head of pancreas	Solitary	Pancreaticoduodenectomy, radiation therapy	72
Bednar et al.^8^	Lobular Phyllodes	75 57	96 48	Jaundice, pain Abdominal pain	Head of pancreas Head of pancreas, lung	Solitary Widespread disease	Pancreaticoduodenectomy Chemotherapy	48 15
Bonapasta et al.^2^	Ductal	51	24	Jaundice, pain	Head of pancreas	Solitary	Pancreaticoduodenectomy	36
Crippa et al.^10^	Lobular Lobular Lobular	46 70 57	60 36 84	Jaundice Jaundice, pain Jaundice, pain	Head of pancreas Head of pancreas Head of pancreas	Solitary Solitary Solitary	Pancreaticoduodenectomy Pancreaticoduodenectomy Pancreaticoduodenectomy	22 38 26
Dar et al.^11^	Ductal	76	108	NR	Pancreas, liver	Widespread disease	Palliative bypass	6
Engel et al.^12^	Signet-ring cells	59	46	Pruritus, choluria	Head of pancreas	Solitary	Palliative bypass, Chemotherapy	15
Estraviz et al.^13^	Ductal	56	36	Jaundice	Head of pancreas	Solitary	Pancreaticoduodenectomy	6 (still alive at the time of report)
Haque et al.^14^	Lobular	85	168	Jaundice, pain	Head of pancreas	Solitary	Palliative bypass	NR
Kita m ura et al.^15^	Ductal	55	117	Jaundice	Head of pancreas	Solitary	Percutaneous drainage	1
Le Borgne et al.^16^	Lobular	48	Synchronous	Jaundice	Head of pancreas	Solitary	Pancreaticoduodenectomy, chemotherapy	12
Mehta et al.^17^	Comedo type	30	36	Jaundice, pruritus	Head of pancreas	Solitary	Pancreaticoduodenectomy, chemotherapy, hormonal therapy	27
Molino et al.^3^	Lobular	68	Synchronous	Jaundice	Head of pancreas	Solitary	Pancreaticoduodenectomy, hormonal therapy	12 (still alive at the time of report)
Mountney etal.^18^	Lobular	57	16	Jaundice	Head of pancreas	Solitary	Palliative bypass, hormonal therapy	24
Moussa et al.^19^	Ductal Lobular	53 35	132 45	Acute pancreatitis Abdominal mass	Head of pancreas Body of pancreas	Solitary Solitary	Radiation therapy, chemotherapy, hormonal therapy Total pancreatectomy, chemotherapy	50 7
Nomizu et al.^20^	Lobular	46	80	Jaundice	Head of pancreas	Solitary	Pancreaticoduodenectomy, chemotherapy, hormonal therapy	18
Odzak et al.^21^	Lobular	48	Synchronous	Jaundice, ascites	Head of pancreas	Widespread disease	Palliative care	NR
Pan et al.^22^	Lobular	59	182	Jaundice	Head of pancreas	Solitary	Chemotherapy, hormonal therapy	21
Pappo et al.^23^	Lobular	52	24	Jaundice	Pancreas, gallbladder	Widespread disease	Palliative bypass, hormonal therapy	16
Pérez Ochoa et al.^24^	Lobular Ductal	60 55	1 108	Jaundice None	Head of pancreas, bone Tail of pancreas	Widespread disease Solitary	Biliary stent, pancreaticoduodenectomy, chemotherapy Distal pancreatectomy, splenectomy, chemotherapy	2 2
Razzetta et al.^25^	Lobular	51	Synchronous	Jaundice, pain, diarrhea	Head of pancreas, bone	Widespread disease	Pancreaticoduodenectomy, neoadjuvant chemotherapy, mastectomy	5
Tohnosu et al.^26^	Scirrhous type	54	52	None	Tail of pancreas	Solitary	Distal pancreatectomy, chemotherapy, hormonal therapy	5
Z’graggen et al.^27^	Lobular	NR	96	Jaundice	Head of pancreas	Solitary	Biliary and gastric bypass, chemotherapy	54
Current study	Ductal	64	24	Pain	Tail of pancreas	Solitary	Distal pancreatectomy, chemotherapy	18 (still alive at the time of report)

NR = not reported.

The clinical signs of this condition are unspecific, with abdominal pain and obstructive jaundice as the main findings.^4^ The absence of characteristic clinical signs and symptoms leads to investigation by means of imaging tests. Ultrasonography, computed tomography and magnetic resonance imaging are frequently used for making this diagnosis; however, the radiological features of primary pancreatic tumors and pancreatic metastases are difficult to differentiate. Use of serum markers such as CA-15.3 may help in making the diagnosis, although in some cases its serum elevation is not relevant.^2^^,^^5^^,^^6^^,^^7^^,^^8^^,^^9^^,^^28^ The most accurate diagnostic method is pancreatic biopsy. Some studies have suggested that fine-needle biopsies guided by endoscopic ultrasound or percutaneously should be used.^3^ The unavailability both of tests for this marker and of endoscopic ultrasound at our service precluded their use in the present case; however, this should not prevent the oncology and surgery teams from recommending operative treatment in cases without widespread disease.

The prognosis for patients with pancreatic metastatic disease is usually better than for patients with primary pancreatic tumors.^2^ Masetti et al. analyzed the prognostic factors relating to metastatic tumors in the pancreas and found two and five-year survival rates of 57.1% and 34.3% in cases of pancreatic metastasis due to breast cancer, respectively.^28^ Surgical resection in cases with disease limited to the pancreas is considered to be the main form of treatment, despite its morbidity.^3^

## CONCLUSION

Based on this study and the evidence available to date, it may be concluded that in cases of pancreatic lesions detected in patients with previous histories of breast neoplasms, the possibility of pancreatic metastasis should be carefully considered.
